# Persistent postmating, prezygotic reproductive isolation between populations

**DOI:** 10.1002/ece3.4441

**Published:** 2018-08-19

**Authors:** Martin D. Garlovsky, Rhonda R. Snook

**Affiliations:** ^1^ Department of Animal and Plant Sciences University of Sheffield Sheffield UK; ^2^ Department of Zoology Stockholm University Stockholm Sweden

**Keywords:** *Drosophila montana*, gametic isolation, postmating prezygotic isolation, sexual conflict, sexual selection, speciation

## Abstract

Studying reproductive barriers between populations of the same species is critical to understand how speciation may proceed. Growing evidence suggests postmating, prezygotic (PMPZ) reproductive barriers play an important role in the evolution of early taxonomic divergence. However, the contribution of PMPZ isolation to speciation is typically studied between species in which barriers that maintain isolation may not be those that contributed to reduced gene flow between populations. Moreover, in internally fertilizing animals, PMPZ isolation is related to male ejaculate—female reproductive tract incompatibilities but few studies have examined how mating history of the sexes can affect the strength of PMPZ isolation and the extent to which PMPZ isolation is repeatable or restricted to particular interacting genotypes. We addressed these outstanding questions using multiple populations of *Drosophila montana*. We show a recurrent pattern of PMPZ isolation, with flies from one population exhibiting reproductive incompatibility in crosses with all three other populations, while those three populations were fully fertile with each other. Reproductive incompatibility is due to lack of fertilization and is asymmetrical, affecting female fitness more than males. There was no effect of male or female mating history on reproductive incompatibility, indicating that PMPZ isolation persists between populations. We found no evidence of variability in fertilization outcomes attributable to different female × male genotype interactions, and in combination with our other results, suggests that PMPZ isolation is not driven by idiosyncratic genotype × genotype interactions. Our results show PMPZ isolation as a strong, consistent barrier to gene flow early during speciation and suggest several targets of selection known to affect ejaculate‐female reproductive tract interactions within species that may cause this PMPZ isolation.

## 1 INTRODUCTION

Speciation requires the accumulation of barriers to gene flow between populations and subsequent taxa. Identifying the barriers that act early during the evolution of reproductive isolation is critical to determine how speciation proceeds (Butlin et al., 2012; Coyne & Orr, 2004; Turelli, Barton, & Coyne, 2001). Reproductive barriers to gene flow can broadly be classified into three categories. Premating reproductive barriers reduce the incidence of hybridization events between taxa (Dopman, Robbins, & Seaman, 2010; Hoskin, Higgie, McDonald, & Moritz, 2005; Lackey & Boughman, 2017; Murray & Clarke, 1980), while postzygotic reproductive barriers are those that result in reduced fitness of hybrid offspring, either due to intrinsic genetic defects (i.e., sterility or inviability), or low fitness in either of the parental habitats (Cooper, Sedghifar, Nash, Comeault, & Matute, 2017; Presgraves, 2010; Wu & Ting, 2004). The third class of reproductive barriers is postmating, prezygotic (PMPZ) reproductive barriers—incompatibilities relating to interactions between the sexes that act after copulation, but before karyogamy—preventing successful fertilization between populations or taxa. Both premating and postzygotic reproductive barriers to gene flow have been studied extensively, however, only relatively recently have PMPZ reproductive barriers begun to be considered in more detail as potentially important reproductive barriers.

The fast‐paced molecular evolution of reproductive tract tissues within populations, accelerated by sexual selection and sexual conflict, is predicted to result in rapid divergence between populations in allopatry and the emergence of PMPZ reproductive incompatibilities between populations early during reproductive isolation (Eady, 2001; Panhuis, Butlin, Zuk, & Tregenza, 2001). In polyandrous mating systems with internal fertilization, where females mate multiply within a single reproductive cycle, the ejaculates of multiple males may overlap within the female reproductive tract. Different males’ ejaculates must then compete to fertilize ova (sperm competition), and females retaining sperm from multiple males may bias paternity (cryptic female choice). Such postcopulatory sexual selection, and its attendant sexual conflict within populations (Andersson, 1994; Andersson & Simmons, 2006; Arnqvist & Rowe, 2002; Gavrilets, 2000), can shape the evolution of intersexual interactions during copulation and fertilization (Bernasconi et al., 2004; Birkhead & Pizzari, 2002; Firman, Gasparini, Manier, & Pizzari, 2017). Rapid evolution of such phenotypes is supported by evidence that genes encoding reproductive tract proteins are among the fastest evolving, showing rapid protein sequence and gene expression evolution (Hollis, Houle, Yan, Kawecki, & Keller, 2014; Perry et al., 2016; Swanson & Vacquier, 2002; Veltsos, Fang, Cossins, Snook, & Ritchie, 2017).

Postmating, prezygotic isolation in external fertilizers is mostly limited to incompatibilities relating to chemo‐attraction between gametes (Weber et al., 2017) and/or gamete interactions at the cell surface (Vacquier & Swanson, 2011). For internal fertilizers, an additional array of potential PMPZ reproductive barriers can act as a result of the complex series of events that take place within the female reproductive tract after mating (Bloch Qazi, Heifetz, & Wolfner, 2003; Orr & Brennan, 2015; Schnakenberg, Siegal, & Bloch Qazi, 2012). In single heterospecific matings, successful fertilization can be decreased or prevented by reduced sperm transfer by males, and/or reduced transport, storage, and viability of hetero‐specific sperm in females (Ahmed‐Braimah, 2016; Kelleher & Markow, 2007; Kohyama, Matsubayashi, & Katakura, 2016; Larson, Hume, Andrés, & Harrison, 2012; Manier et al., 2013; Reinhardt, 2006; Rose, Brand, & Wilkinson, 2014). PMPZ isolation has also been suggested to occur when hetero‐specific matings result in reduced egg production compared to con‐specific matings, even though fertilization is successful (e.g., Matute & Coyne, 2010; Turissini, McGirr, Patel, David, & Matute, 2018). PMPZ isolation in internally fertilizing animals may also be manifested only when con‐ and hetero‐specific ejaculates are in competition. For instance, con‐specific sperm precedence occurs when paternity is biased to sperm from the con‐specific male even though hetero‐specific male sperm may fertilize ova in single matings (Castillo & Moyle, 2014; Cramer, Ålund, McFarlane, Johnsen, & Qvarnström, 2016; Price, 1997; Yeates et al., 2013).

A growing body of literature now shows PMPZ isolation is the primary or only barrier to gene flow in some closely related taxa, suggesting an important role in the early evolution of reproductive isolation (Ahmed‐Braimah, Unckless, & Clark, 2017; Bono, Matzkin, Hoang, & Brandsmeier, 2015; Cramer et al., 2016; Dean & Nachman, 2009; Soudi, Reinhold, & Engqvist, 2016; Turissini et al., 2018). However, the majority of research has focused on incompatibilities arising between species even though barriers that maintain reproductive isolation after divergence may not be the same barriers that were important in reducing gene flow during the initial stages of the speciation process (Butlin et al., 2012; Coyne & Orr, 2004; Turelli et al., 2001). Therefore, to understand the factors important during the initial stages of divergence, more focus is needed on the reproductive barriers acting between recently diverged populations of the same species.

*Drosophila montana* provides an opportunity to study the role of PMPZ isolation and the early stages of the speciation process. This species is distributed across the northern hemisphere at high altitudes and latitudes with a well‐documented ecology and phylogeographic history (Aspi, Lumme, Hoikkala, & Heikkinen, 1993; Mirol et al., 2007). Investigating the contribution of both pre‐ and postmating reproductive barriers between three *D. montana* populations, two from North America, and one from Europe, found hybrid crosses between populations exhibited PMPZ isolation (Jennings, Snook, & Hoikkala, 2014). PMPZ isolation was a consequence of sperm failing to penetrate eggs, even though motile sperm were transferred by males and stored by females (Jennings et al., 2014). These populations also exhibited premating isolation which increased with genetic distance, suggesting isolation by distance; however, there was no clear relationship between genetic distance and the strength of PMPZ isolation. While premating reproductive barriers to gene flow are undoubtedly important in these populations, strong PMPZ isolation that is not associated with isolation by distance suggests PMPZ isolation may be especially important early during the evolution of reproductive isolation.

Yet, there remain several open questions about the evolution of PMPZ isolation, both specifically for this system and generally. Are patterns of PMPZ isolation unique to these populations or more widespread? Are PMPZ isolation patterns repeatable with individuals tested from the same location but collected at different times? Do all individuals show similar strengths of PMPZ isolation or is PMPZ isolation idiosyncratic between some individuals? Additionally, male and female mating history may also influence the expression and strength of PMPZ isolation with consequences for the importance of PMPZ isolation in limiting gene flow between populations. Mating history is known to have both ameliorating and exacerbating effects on other types of reproductive incompatibility, such as cytoplasmic incompatibility between *Wolbachia*‐infected *D. simulans* males and uninfected females which is ameliorated if males have remated frequently (Awrahman, Champion de Crespigny, & Wedell, 2014; Karr, Yang, & Feder, 1998). In contrast, receipt of multiple foreign ejaculates by females may amplify infertility due to receipt of toxic foreign ejaculates (Kelleher & Markow, 2007; Knowles & Markow, 2001). Do male and female mating history influence the strength of PMPZ isolation?

We addressed these outstanding questions about the evolution of PMPZ isolation by testing both recent collections from the same North American locations as previously described and additional new populations. We also assessed whether PMPZ isolation is acting at the population level or only between specific genotype × genotype interactions from different populations. Furthermore, we determined whether the presence and strength PMPZ isolation are affected by intrinsic infertility or male and female mating history.

## 2 METHODS

### 2.1 Fly stocks

**Figure 1:**
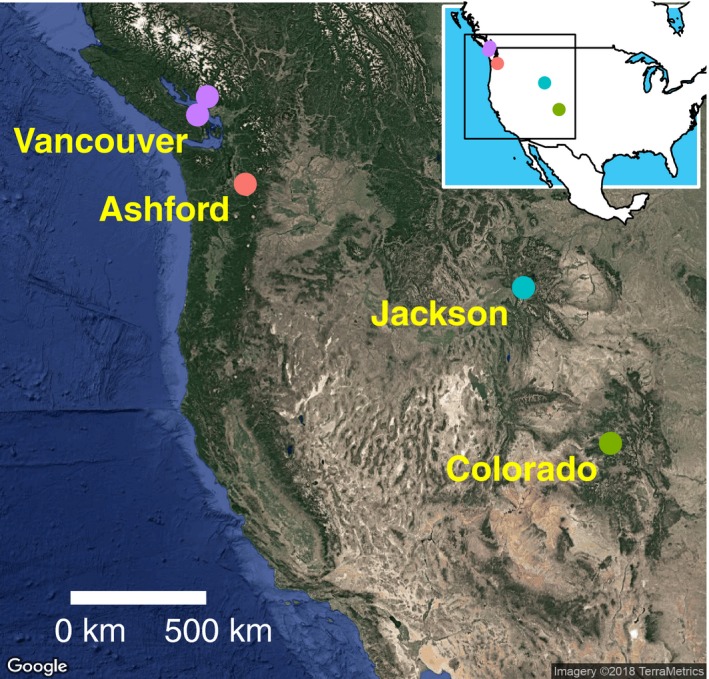
Collection locations of *Drosophila montana* populations. Maps created using the “ggmap” package in R (Kahle & Wickham, 2013) [Colour figure can be viewed at http://wileyonlinelibrary.com]

Adult *D. montana* were collected from riparian habitats using malt bait buckets and mouth aspirators from Ashford, Washington, USA, in 2013 (referred to as Ashford, and abbreviated as A); Crested Butte, Colorado, USA in 2009 and 2013 (referred to as Colorado, abbreviated as C); Jackson, Wyoming, USA in 2013 (referred to as Jackson; abbreviated as J); and Vancouver, British Columbia, Canada (referred to as Vancouver; abbreviated as V) in 2008 and 2014 (Figure 1, Table S1). Both iso‐female lines and population cages were tested for PMPZ isolation. Population cages for Colorado (2013) and Vancouver (2008) were established by combining 20 F3 progeny of each sex from each of 20 iso‐female lines. The population cage for Vancouver (2014) was established in the same way except F4 progeny from 21 iso‐female lines were merged. All populations and iso‐female lines were cultured in the laboratory on Lakovaara malt medium (Lakovaara, 1969) in overlapping generations at 19°C in constant light (Jennings et al., 2014). Flies used for experimentation were collected within 3 days of eclosion, as male reproductive maturity does not occur until at least 8 days posteclosion (Pitnick, Markow, & Spicer, 1995). All experiments were carried out using flies aged between 21 and 28 days from eclosion. In each experiment, we carried out all four possible crosses between the two focal populations being tested, where the female population is always indicated first (e.g., AA is a cross between Ashford females and Ashford males and AC is a cross between Ashford females and Colorado males).

### 2.2 Statistical analysis

We outline specific statistical tests for each experiment and trait we analyze at the end of each section (described below). All statistical analyses were performed in R (version 3.3.0) (R Core Team, 2016). Generalized linear mixed effects models (GLMMs) and parametric bootstrap simulations to obtain model predicted values (±95% confidence intervals) were fitted using the “lme4” package (Bates, Mächler, Bolker, & Walker, 2015). We tested for significance of fixed effects and interactions via likelihood ratio tests (LRT), or parametric bootstrapped simulations using the *PBmodcomp* function from the “pbkrtest” package (Halekoh & Højsgaard, 2014). When necessary, we performed post hoc Tukey's honest significant difference (HSD) tests using the *glht* function from the “multcomp” package (Hothorn, Bretz, & Westfall, 2008).

### 2.3 Postmating, prezygotic isolation between North American populations of *D. montana*

To test the pattern of PMPZ isolation previously reported (Jennings et al., 2014) with a new Colorado population and to identify other populations showing evidence of PMPZ isolation, we performed a series of crosses between Colorado and Vancouver, and two previously untested populations—Ashford and Jackson. PMPZ isolation is measured by egg hatch success (number of eggs oviposited that hatched) and/or number of progeny produced. For each pair of focal populations, we performed fully factorial experiments, generating data from both parental crosses, and the two reciprocal between‐population crosses. We refer to the four crosses within each pair‐wise comparison as the cross‐type. Final sample sizes and details of the specific strain x strain cross‐types performed in each of the pair‐wise combinations between the four populations, and a summary of PMPZ outcomes are presented in Figures S1 and S3.

For each cross, we assessed PMPZ isolation by mating single virgin males and females (*n* = 30 per cross‐type per block). Note all crosses were observed for mating over a 4‐hr period to exclude confounding sources of reproductive isolation. If mating did not occur within this timeframe, then we discarded that pair. If mating occurred, then the pair was mouth aspirated into a chamber of an oviposition “manifold” after mating (Jennings et al., 2014). Manifolds were connected to oviposition plates containing a molasses‐agar egg laying medium with a drop of dried yeast paste added and incubated at 19°C. Females were left to oviposit for 2 days, before changing the oviposition plate, and allowing a further 2 days of oviposition. Following the second 2‐day oviposition period, flies were discarded, the numbers of eggs laid were counted (fecundity), and the oviposition plate returned to the incubator. Two days later, the numbers of unhatched eggs on the second oviposition plate were counted again. Females that did not oviposit were excluded from analyses.

To assess differences between cross‐types in fecundity, we fitted GLMMs with Poisson errors and a log link, using the total number of eggs laid as the response variable. To assess differences between cross‐types in hatching success rates, we fitted GLMMs with binomial errors and a logit link, using the numbers of hatched eggs (“successes”) and unhatched eggs (“failures”) as the response variable. All models included cross‐type as the only fixed effect, and we performed analyses on each of the six crosses separately. We included random effects for the specific strains tested from within each population, and for experimental block, to account for variation between strains tested in each cross‐type, and variation between blocks testing each cross between populations, respectively. All models also included an observation level random effect (OLRE) to account for overdispersion (Harrison, 2014, 2015). To test whether there was a significant effect of cross‐type on each measure of PMPZ isolation, we compared each model to a null model including the global intercept (~1) as the only fixed effect (but with the same random effects structure), with 10,000 parametric bootstrapped simulations.

#### 2.3.1 Postmating, prezygotic isolation mechanism

Previous work showed that PMPZ isolation was manifested by the lack of fertilization, despite males transferring and females storing motile sperm (Jennings et al., 2014). To confirm that egg hatching failure was due to lack of fertilization in additional crosses and populations, eggs from a subset of crosses were scored for development following the same protocol as previously used (Jennings et al., 2014). Briefly, for each of the four cross‐types in the Colorado 2013–Jackson 2013 and Colorado 2013–Vancouver 2008 cross (Table S1), we mouth aspirated flies (30–40 of each sex) into half‐pint bottles covered with an oviposition plate, containing molasses‐agar egg laying medium with a drop of dried yeast paste added. Oviposition plates were replaced every 24 hr, and eggs were collected *en masse*, fixed, and stained using DAPI. Eggs were inspected using fluorescence microscopy to score for development. Nondeveloping eggs were further inspected using differential interference contrast microscopy to score eggs for presence or absence of sperm in the egg, indicating whether fertilization had been successful. We tested for differences in the numbers of fertilized eggs in each cross‐type using Pearson's chi‐squared test. Note that fertilization failure cannot be due to cytoplasmic incompatibility as a consequence of *Wolbachia* infection in our stocks because we found no visual evidence of *Wolbachia* (Stouthamer, Breeuwer, & Hurst, 1999) and previous analyses investigating *Wolbachia* prevalence across the *Drosophila* phylogeny found no molecular evidence of *Wolbachia* in the *virilis* group (Bourtzis, Nirgianaki, Markakis, & Savakis, 1996; Mateos et al., 2006).

### 2.4 Testing intrinsic male infertility

To assess whether low fertilization success in between‐population crosses could be confounded by poor male fertility irrespective of the identity of his mate, we mated focal males to both a between‐ and within‐population female. For this experiment, we used flies from the Colorado 2013 and Vancouver 2008 population cages (Table S1). Focal males were paired individually with two virgin females on consecutive days, one within‐ and one between‐population female. To account for any mating order effects, we randomly assigned half of males (*n* = 20 per cross‐type) to have a between‐population female as the first mate, and the other half of males, a within‐population female as the first mate. All matings were observed; if mating did not occur, pairs were discarded. Mated females were mouth aspirated singly to a manifold chamber after mating and data collected for hatching success, as described above. Males were transferred to new vials containing malt medium and mated the next day with the other female. Second females were mouth aspirated singly to a manifold chamber after mating and data collected for hatching success, as described above. To test whether males with low fertilization success in between‐population crosses also had low within‐population fertilization success, we calculated Spearman's rank correlation coefficient for the proportion of eggs hatching between males’ first and second mating, for each set of males separately (i.e., Colorado males having a between‐population partner first or second, Vancouver males having a between‐population partner first or second).

### 2.5 Consistency of postmating, prezygotic isolation across different genotypes

Reproductive incompatibility could be the result of idiosyncratic genotype × genotype interactions between males and females from different populations, rather than a population‐level effect. To assess whether specific female genotype × male genotype interactions yielded variable fertilization outcomes, we used matings within and between—individuals from the Colorado 2013 and Vancouver 2008 population cages (Table S1). Focal males (*n* = 10 per cross‐type) were paired individually with a virgin female and monitored for mating. Mated females were mouth aspirated singly to a manifold chamber after mating, while males were transferred to new vials containing malt medium. The next day, focal males were presented with another virgin female from the same population as on the previous day. Mated females were mouth aspirated singly to a manifold chamber after mating. We repeated this for five consecutive days. Mated females were processed for egg hatch success as previously described. To assess the between individual variance in hatching success, for those males that mated three or more times, we fitted a GLMM with binomial errors and a logit link, using egg hatch success (i.e., counts of hatched and unhatched eggs) as the response variable and mating day as the only fixed effect. We fitted a model for each cross‐type separately, as combining groups across the different cross‐types would artificially inflate the between‐group variance. Models included a random effect for male identify and an OLRE.

### 2.6 Effects of male multiple mating on postmating, prezygotic isolation

To assess the effect of multiple mating on male fertilization success, we used the data collected from “Consistency of postmating, prezygotic isolation across different genotypes” above, and fitted a GLMM with binomial errors and a logit link, using egg hatch success (i.e., counts of hatched and unhatched eggs) as the response variable, with cross‐type, mating number, and the cross‐type × mating number interaction as fixed effects and male identity as a random effect, and an OLRE.

### 2.7 Effects of female multiple mating on postmating, prezygotic isolation

To test whether multiple insemination affected the strength of PMPZ isolation, we mated focal females to multiple males. For each of the four cross‐type combinations between the Colorado 2013 and Vancouver 2008 population cages (Table S1), focal females (*n* = 15 per cross‐type) were paired individually with a virgin male. Males were discarded immediately after mating. The next day, focal females were mouth aspirated into a new vial housing a virgin male from the same population as on the previous day. We repeated this for five consecutive days. Only females who mated on three or more consecutive days were kept for analysis. All progeny eclosing from each oviposition vial were subsequently counted and sexed. To test the effect of multiple insemination on the total strength of PMPZ isolation, we fitted a GLMM with Poisson errors, using the total number of progeny enclosed as the response variable, with cross‐type, mating number, and the cross‐type × mating number interaction as fixed effects, and a random effect for female identity, and an OLRE.

## 3 RESULTS

### 3.1 Postmating, prezygotic isolation between North American populations of *D. montana*

We performed a series of pair‐wise fully factorial crosses between four *D. montana* populations from across North America to identify populations showing evidence of PMPZ isolation. Previous studies have included reduced female fecundity following mating with a foreign male as a PMPZ reproductive barrier (Matute, 2010; Matute & Coyne, 2010; Turissini et al., 2018). Here, we found a significant effect of cross‐type on female fecundity in three (Ashford‐Jackson, Ashford‐Vancouver and Jackson‐Vancouver crosses) of the six pair‐wise population crosses (Table 1). However, these responses were asymmetric and not in the predicted direction if PMPZ isolation was acting (Figures S1 and S2). In Ashford‐Jackson crosses, one between‐population cross had greater fecundity than both the reciprocal cross and one of the parental crosses; Jackson males elevated Ashford female fecundity above that of the reciprocal cross (AJ vs. JA; Tukey's HSD; *p* = 0.013; Table S4) and the within‐population Ashford cross (AJ vs. AA; Tukey's HSD, *p* = 0.019; Table S4). The same pattern was found for the Ashford‐Vancouver cross; Ashford males elevated Vancouver female fecundity above the reciprocal cross (VA vs. AV; Tukey's HSD, *p* = 0.026; Table S5) and the within‐population Vancouver cross (VA vs. VV; Tukey's HSD, *p* = 0.003; Table S5). In the Jackson‐Vancouver cross, between‐population crosses differed from each other, but not from either within‐population cross (JV had lower fecundity than VJ (Tukey's HSD; *p* = 0.007; Table S8) but these did not differ from parental crosses). Moreover, these pair‐wise population comparisons showed no effect of cross‐type on hatching success (all *p* > 0.06, Table 1; Figures S3 and S4). Thus, there is no evidence of PMPZ isolation between these three populations.

**Table 1:** Measures of postmating, prezygotic isolation (fecundity and hatching success) between North American populations of *Drosophila montana*

	Cross											
	A × C (3)		A × J (1)		A × V (3)		C × J (1)		C × V (7)		J × V (1)	
Measure of PMPZ	χ^2^	*p*	χ^2^	*p*	χ^2^	*p*	χ^2^	*p*	χ^2^	*p*	χ^2^	*p*
Fecundity	2.96	0.590	**11.56**	**0.015**	**10.14**	**0.050**	3.26	0.366	7.31	0.144	**10.20**	**0.023**
Hatch success	**21.74**	**0.005**	1.06	0.802	2.52	0.690	**107.08**	**<0.001**	**39.571**	**<0.001**	7.76	0.062

*Note*. *p*‐Values obtained from 10,000 parametric bootstrap simulations, comparing the model including cross‐type as the only fixed effect against the null (intercept only) model. Cross lists the two populations being fully reciprocally crossed (e.g., A × C = AA, AC, CA, CC where A, Ashford; C, Colorado; J, Jackson; V, Vancouver; the population of the female is listed first). Because each cross contained all four cross‐types for each measure of PMPZ, *df* = 3 for all models. Number in parentheses after the cross is the number of replicate blocks. Total sample sizes for each cross provided in Figure 2.

Bold values indicate significance of <0.05.

**Figure 2:**
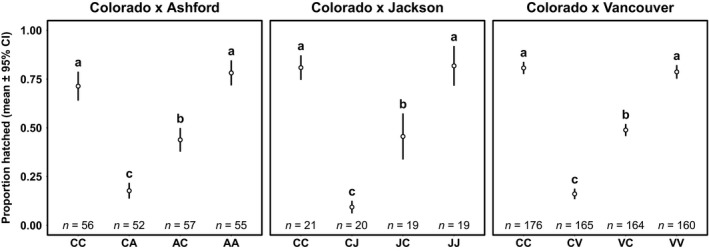
Proportion of eggs hatching (mean ± 95% confidence intervals) in crosses involving Colorado. Within each panel, different letters indicate significant differences from post hoc Tukey's HSD. Letters are recycled in each panel; however, supplementary analyses showed that letters shared across panels also represent statistically equivalent groups (see Section 3). Cross‐types are abbreviated with the female population given first. A, Ashford; C, Colorado; J, Jackson; V, Vancouver. *N* = number of mating pairs over all experimental blocks. N.B. crosses not showing PMPZ isolation are shown in Figure S4

The three pair‐wise population comparisons involving the Colorado population showed no difference in fecundity between cross‐types (Table 1). However, there was a significant effect of cross‐type on hatching success (Figure 2; Table 1). Hatching success was high and similar (≥75%) for within‐population crosses (Tukey's HSD; all *p* > 0.5; Tables S4, S6, and S7), whereas the reciprocal between‐population crosses were all significantly different from both within‐population crosses, and from each other (Figure 2; Tukey's HSD; all *p* < 0.003; Tables S4, S6, and S7). Colorado females mated to a foreign male had <20% hatching success and, in the reciprocal crosses; foreign females mated to Colorado males had ~50% hatching success (Figure 2). In summary, crosses that involved flies from Colorado exhibited asymmetrical PMPZ isolation with all three other populations tested. In contrast, the crosses between pairs of those three populations showed no evidence of PMPZ isolation (Figures S3 and S4).

To determine whether the strength of PMPZ isolation with the Colorado population depended on the non‐Colorado population, we tested whether there was a significant difference in hatching success by pooling data across all experimental blocks for all between‐population crosses involving only Colorado females or Colorado males (i.e., incompatible crosses). Colorado females showed equally low hatching success, regardless of the origin of the between‐population male (LRT = 0.99, *df* = 2, *p* = 0.610). Likewise, Colorado males showed equivalently low hatching success, regardless of the origin of the between‐population female (LRT = 1.99, *df* = 2, *p* = 0.372). To determine whether egg hatch success varied between compatible crosses, we pooled data across all experimental blocks but excluded all between‐population crosses involving both Colorado males and Colorado females. Compatible crosses showed high hatching success that did not differ between crosses (LRT = 7.95, *df* = 9, *p* = 0.539). In summary, the strength of PMPZ isolation involving Colorado was equal across all populations, regardless of the population origin of the foreign mating partner (see legend in Figure 2) whereas all other between‐population crosses had hatching success equivalent to within‐population success (Figure S4).

#### 3.1.1 Postmating, prezygotic isolation mechanism

**Figure 3:**
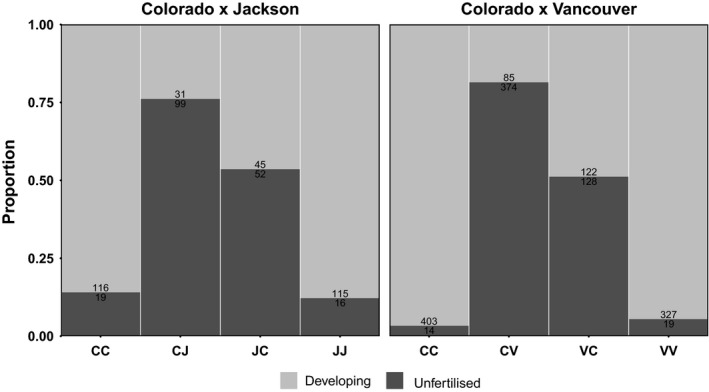
Proportion of developing (light gray) and unfertilized (dark gray) eggs in each cross‐type. Cross‐types are abbreviated with the female population given first. C, Colorado; J, Jackson; V, Vancouver. Numbers in bars indicate the total number of eggs counted

After surveying all populations for evidence of PMPZ isolation, we scored oviposited eggs for development and fertilization status in the Colorado 2013–Vancouver 2008 cross to confirm low hatching rates were due to the same pattern of fertilization failure previously reported (Jennings et al., 2014). We also scored eggs from the Colorado 2013–Jackson 2013 cross to confirm whether this was a consistent PMPZ isolating mechanism. We found a significant effect of cross‐type on the number of eggs fertilized in the Colorado‐Vancouver (*χ* ^2^ = 766.55, *df* = 3, *p* < 0.001) and in the Colorado‐Jackson (*χ* ^2^ = 160.56, *df* = 3, *p* < 0.001) crosses. While most eggs were developing in all within‐population crosses, eggs oviposited by Colorado females mated to foreign males had <25% of eggs fertilized, and foreign females mated to Colorado males had <50% of eggs fertilized (Figure 3).

### 3.2 Testing intrinsic male infertility

To test whether reduced fertilization success could be due to intrinsic male infertility, we calculated Spearman's rank correlation coefficient for the proportion of eggs that hatched for males mated to both a within‐ and between‐population female (Table 2, Figure S5). There was no correlation in the level of fertility between the first and second mating, regardless of mating order (Spearman's rank correlation, all *p* > 0.48, Table 2). Therefore, hatching success rates can be attributed to the cross‐type alone and are not confounded by male infertility.

**Table 2:** Spearman's rank correlation coefficients calculated for the proportion of eggs hatching for males mated to virgin within‐ and between‐population females

Male population	Female population		*N*	Spearman's rho	*p*
	First mating	Second mating			
Colorado	Colorado	Vancouver	20	−0.032	0.896
	Vancouver	Colorado	18	0.176	0.482
Vancouver	Vancouver	Colorado	18	0.003	0.990
	Colorado	Vancouver	19	0.093	0.713

### 3.3 Consistency of postmating, prezygotic isolation across different genotypes

To test whether PMPZ isolation was due to either specific female genotype × male genotype interactions or a population‐level phenomenon, in the Colorado 2013–Vancouver 2008 population cage cross (Table S1), we assessed the between individual variance in hatching success for males that mated at least three times over consecutive days (most males mated the maximum of 5 times; median number of mates = 5). Half of males were mated to virgin females from their own population, and the other half of males were mated to foreign females, and we modeled each cross‐type separately to properly partition between‐group variance. In all cases, estimates of between individual variance (male identity random effect variance) were 0 signifying inclusion of the male identity random effect was not warranted in the models, and models including male identity as a random effect had higher AIC_c_ scores than those without (Table S9). Thus, between male variance in hatching success was negligible, indicating a consistent pattern of PMPZ isolation acting across a range of female × male genotype interactions between populations.

### 3.4 Effects of male and female multiple mating on postmating, prezygotic isolation

**Figure 4:**
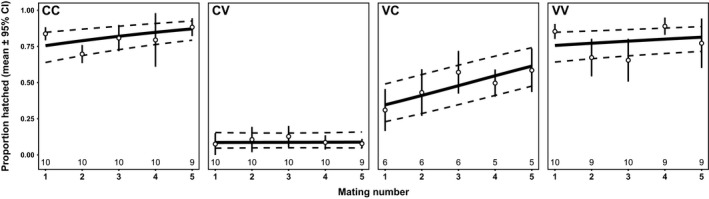
Proportion of eggs hatching (mean and model predicted values ± 95% CI) per day for males mated to between three and five within‐ or between‐population females over consecutive days. Cross‐types are abbreviated with the female population given first. C, Colorado; V, Vancouver. Numbers below points indicate sample sizes (number of mating pairs each day)

To test whether male multiple mating affected the strength of PMPZ isolation, in the Colorado 2013–Vancouver 2008 population cage cross (Table S1), we assessed the effect of multiple mating of males on fertilization success (Figure 4). There was a significant effect of cross‐type (LRT = 106.08, *df* = 3, *p* < 0.001; incompatible crosses had low egg hatch success) and a marginally significant effect of mating number on egg hatch success (LRT = 9.323, *df* = 4, *p* = 0.054) suggesting that males improve fertilization success as they mate more. The cross‐type × mating number interaction was not significant (LRT = 2.29, *df* = 3, *p* = 0.514). Thus, there was no effect of male mating history on the strength of PMPZ isolation.

**Figure 5:**
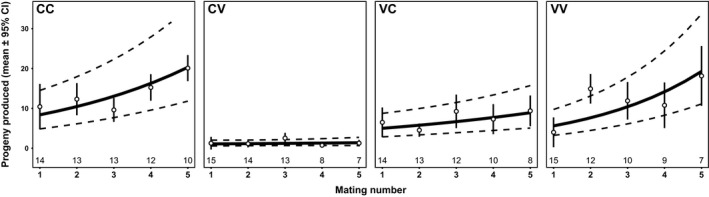
Per‐day progeny production (mean and model predicted values ± 95% CI) for females mated to multiple within‐ or between‐population males over consecutive days. Cross‐types are abbreviated with the female population given first. C, Colorado; V, Vancouver. Numbers below points indicate sample sizes (number of mating pairs each day)

We also tested the effect of females receiving multiple ejaculates on the strength of PMPZ isolation, by counting the total number of adult progeny produced each day by females inseminated by up to five males. As with males, almost every female mated every day (median number of mates = 5). Like males, we found a significant effect of cross‐type (LRT = 58.57, *df* = 6, *p* < 0.001; incompatible crosses had low fertility). Unlike males, we saw a strong effect of mating number of progeny production per day (LRT = 24.36, *df* = 4, *p* < 0.001); the rate of progeny production increased with mating number similarly in all four crosses (Figure 5). However, we still found no effect of the cross‐type × mating number interaction (LRT = 2.71, *df* = 3, *p* = 0.438), thus, there was no effect of female mating history on the strength of PMPZ isolation.

## 4 DISCUSSION

Identifying early acting reproductive barriers is central to understanding the factors that contribute to the initial stages of the speciation process. While recent efforts have increasingly identified PMPZ isolation as critical in these early stages (Devigili et al., 2018; Soudi et al., 2016; Turissini et al., 2018), outstanding questions remain about factors that could influence the extent of gene flow between populations exhibiting PMPZ isolation. We addressed the repeatability and consistency of PMPZ isolation acting between different populations, the mechanism of PMPZ isolation, and how male and female mating history influences the strength of PMPZ incompatibility. We found a recurrent and robust pattern of PMPZ isolation between *D. montana* populations. Crosses involving either males or (particularly) females from Colorado exhibited PMPZ isolation with three other populations, while crosses between those three populations remained fertile with each other. Incompatibility was due to fertilization failure but was not a consequence of intrinsic male infertility. As reproductive isolation is not complete between these populations, incompatibilities may only be present between specific female × male genotype interactions, but we found no variation in hatching success attributable to male identity. Thus, we show that PMPZ isolation, at least between Colorado and Vancouver, is acting at the population level. Multiple mating by males did not influence fertilization competency; compatible crosses remained compatible and incompatible crosses remained incompatible. Likewise, while multiple insemination of females increased the number of progeny produced per day in all crosses, incompatible crosses still produced significantly fewer progeny compared to within‐population crosses. Thus, male or female multiple mating neither exacerbated nor ameliorated incompatibility. These patterns suggest that gene flow will be limited between Colorado individuals and the other populations, at least under these conditions.

Other studies of PMPZ isolation between species have suggested that reduced fecundity is a PMPZ reproductive barrier, even if fertilization occurs normally (Matute, 2010; Turissini et al., 2018). Here, we show that in some between‐population crosses, fecundity is the same as at least one of the parental crosses so PMPZ isolation is not due to a reduction in fecundity. Instead, PMPZ isolation is manifested as a consequence of reduced fertilization rates. For normal and efficient fertilization, a coordinated series of ejaculate‐female reproductive tract interactions are required (Avila, Sirot, LaFlamme, Rubinstein, & Wolfner, 2010; Bloch Qazi et al., 2003; Mattei, Riccio, Avila, & Wolfner, 2015; Pitnick, Wolfner, & Suarez, 2009; Wolfner, 2009). The emergence of PMPZ reproductive barriers may be due to mismatched ejaculate‐female reproductive tract interactions, deriving from population differentiation arising from selection and/or genetic drift. However, Jennings et al. (2014) found no relationship between genetic distance and the strength of PMPZ isolation, suggesting divergence is not simply a result of isolation by distance. Instead, PMPZ isolation likely emerges as a by‐product of both sexual selection and sexual conflict which are important in shaping the rapid co‐evolution of ejaculate‐female reproductive tract interactions (Ahmed‐Braimah et al., 2017; Bono et al., 2015; Mendelson, Martin, & Flaxman, 2014; Pitnick et al., 2009). Given that *D. montana* males transfer and females store motile sperm for fertilization but (most of) these sperm do not penetrate eggs, incompatibility is likely because of mismatches between sperm and egg release. These incompatibilities may arise due to variation between populations in seminal fluid proteins (Sfps) in the male ejaculate that cause profound behavioral, morphological, and physiological changes in the mated female (Avila, Ravi Ram, et al., 2010; Perry, Sirot, & Wigby, 2013; Pitnick et al., 2009; Ravi Ram & Wolfner, 2007; Wolfner, 2009). Candidate Sfps include sex peptide (SP) which binds to the female sex peptide receptor (SPR) in the mated female and is essential for proper release of sperm from storage to ensure efficient fertilization in *D. melanogaster* (Avila, Mattei, & Wolfner, 2015; Avila, Ravi Ram, Qazi, & Wolfner, 2010) and/or Acp36DE and ovulin which are required for efficient sperm storage and oocyte release in *D. melanogaster* (Avila & Wolfner, 2009; Mattei et al., 2015). Future work should examine population variation in *D. montana* Sfp composition to test their potential role in mediating PMPZ isolation and to identify underlying “speciation genes” (Butlin et al., 2012; Nosil & Schluter, 2011; Presgraves, 2010).

Reproductive incompatibility in this system is asymmetrical, which may also help to understand the evolution of ejaculate‐female reproductive tract interactions and the emergence of PMPZ reproductive barriers. Fertilization was reduced more in crosses involving Colorado females (<20% of eggs hatched) than in crosses involving Colorado males (ca. 50% of eggs hatched). Asymmetries in reproductive barriers could result from differences between populations in the strength of sexual selection (Boughman, Rundle, & Schluter, 2005) and the action of sexual conflict (Arnqvist, Edvardsson, Friberg, & Nilsson, 2000). For example, considering the male ejaculate as a polygenic trait, in populations where females have evolved preferences for high trait values of males, females will impose stronger selection on males, thus, reproductive isolation will be stronger in crosses involving those females. However, females from a population where trait values are lower on average, may still accept males (from another population) having higher trait values (Boughman et al., 2005), generating asymmetries in reproductive isolation. Such asymmetries generate predictions to test in future research; if postmating sexual selection is stronger in the Colorado population, then Colorado males should have more competitive and/or otherwise preferred ejaculates than Vancouver males. While a previous study did not find PMPZ isolation between these two populations under a sperm competitive scenario (Ala‐Honkola, Ritchie, & Veltsos, 2016), the Colorado population they used had very low within‐population fertilization success and subsequently went extinct in the laboratory, suggesting some kind of inbreeding depression. Our current research shows recurrent, strong PMPZ isolation between these populations that is not dependent on a particular collection from a particular time and we conclude that PMPZ isolation occurs consistently between these populations (see also Moorhead, 1954).

Reproductive isolation is not complete between the Colorado population and any of the others we tested it against, so it was important to establish whether PMPZ isolation was an interaction between specific female × male genotypes or a more widespread pattern acting across a range of genotypes. We tested focal males against multiple incompatible females and found little between‐male variance in fertilization success, which did not warrant including male identity in the model. Low between‐male variance in fertilization success indicates PMPZ isolation was acting consistently across the range of genotype × genotype interactions tested and was present at the population level. It may be that genotypes were limited after being in culture for a period of time; however, we observe high fertilization success in within‐population crosses suggesting no inbreeding depression. Moreover, our results between Colorado and Vancouver populations were similar regardless of which Colorado and Vancouver populations/iso‐female lines were being tested (this study and Jennings et al., 2014). Even if genetic variability has been eroded during the course of laboratory culture, then this means that alleles of large effect are likely fixed within populations, making future studies identifying speciation genes/loci causing PMPZ isolation easier to detect.

Male and female mating history is known to influence the extent of reproductive incompatibility, which could then influence the strength of PMPZ isolation. For example, Sfps are harmful to females (Chapman, Liddle, Kalb, Wolfner, & Partridge, 1995; Sirot, Wong, Chapman, & Wolfner, 2015; Wolfner, 2009) and foreign seminal fluids may be even more so (Kelleher & Markow, 2007; Knowles & Markow, 2001), thus, multiple mating by females may increase reproductive incompatibility. Males can also modify ejaculate composition depending on whether a female is virgin or mated (Sirot, Wolfner, & Wigby, 2011), which may elicit different effects on female postmating physiology including fertilization efficiency. However, we found no interaction between cross‐type and mating number for either sex, indicating consistent intrinsic incompatibilities between populations. Both male and, to a greater extent, female reproductive success was increased by multiple mating, but this increase was the same relative amount for all crosses. This could be due to several different mechanisms such as females becoming more efficient fertilizers as they age, increased sperm viability and/or sperm number, and ejaculate composition modification.

In summary, we focussed on recently diverged populations of the same species to better understand PMPZ reproductive barriers that could act at the very earliest stages of the speciation process (Butlin et al., 2012; Servedio & Boughman, 2017; Shaw & Mullen, 2011; Tinghitella et al., 2017), the extent to which these barriers are consistent between populations collected at different times and between different genotypes, and how mating histories of the sexes influenced the strength of PMPZ isolation. While there is no guarantee that these populations will continue along the speciation process, we showed consistent, persistent, and reproducible isolation between *D. montana* populations that is manifested at the population level and not influenced by either male or female mating history. PMPZ isolation was asymmetrical and occurred between Colorado individuals crossed with all other tested populations and was a consequence of fertilization failure, likely due to mismatches between ejaculate‐female reproductive tract interactions. Future work will determine the nature of these mismatches and aim to identify the loci contributing to PMPZ isolation.

## CONFLICT OF INTEREST

None declared.

## AUTHOR CONTRIBUTIONS

MDG collected the data and performed analyses. MDG and RRS designed the experiments and wrote the manuscript.

## DATA ACCESSIBILITY

The final dataset is available on Dryad Digital Repository: https://doi.org/10.5061/dryad.6n4g650.
