# Supplementary material for: Persistent postmating, prezygotic reproductive isolation between populations
Source: Ecol Evol. 2018 Aug 19;8(17):9062–73. doi: 10.1002/ece3.4441 (PMC6157668; doi:10.1002/ece3.4441)
Supplement: Supplementary file 1 [file ECE3-8-9062-s001.pdf]

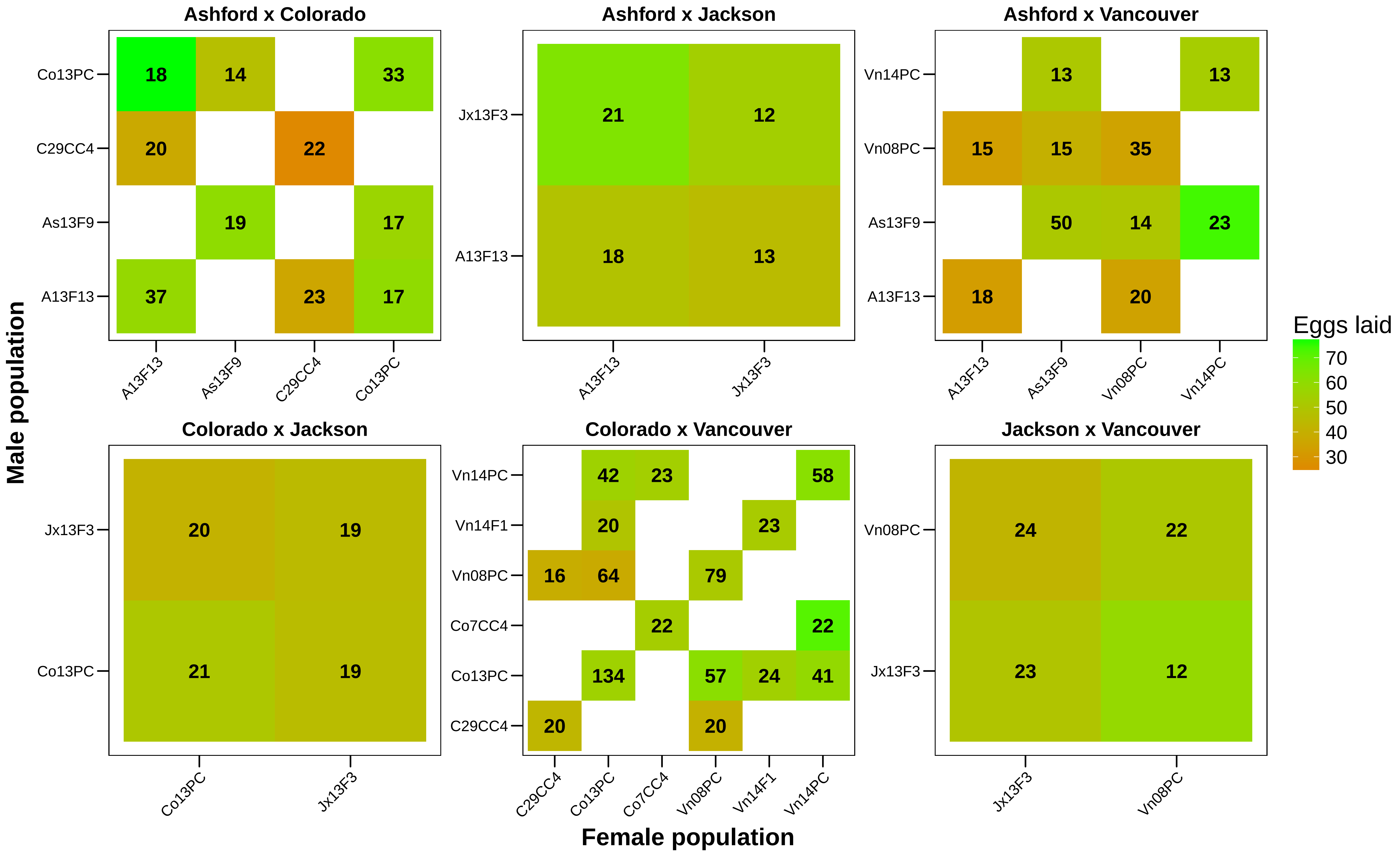

Ashford x Colorado
Ashford x Jackson
Ashford x Vancouver
Colorado x Jackson
Colorado x Vancouver
Jackson x Vancouver
Co13PC
C29CC4
As13F9
A13F13
18
14
33
20
22
19
17
37
23
17
Jx13F3
A13F13
21
12
18
13
Vn14PC
Vn08PC
As13F9
A13F13
13
13
15
15
35
50
14
23
18
20
Vn14PC
Vn14F1
Vn08PC
Co7CC4
Co13PC
C29CC4
42
23
58
20
23
16
64
79
22
22
134
57
24
41
20
20
Jx13F3
Co13PC
20
19
21
19
Vn08PC
Jx13F3
24
22
23
12
A13F13
As13F9
C29CC4
Co13PC
A13F13
Jx13F3
A13F13
As13F9
Vn08PC
Vn14PC
Co13PC
Jx13F3
C29CC4
Co13PC
Co7CC4
Vn08PC
Vn14F1
Vn14PC
Jx13F3
Vn08PC
Eggs laid
70
60
50
40
30
Male population
Female population
