# Supplementary figures and images for: Persistent postmating, prezygotic reproductive isolation between populations
Source: Ecol Evol. 2018 Aug 19;8(17):9062–73. doi: 10.1002/ece3.4441 (PMC6157668; doi:10.1002/ece3.4441)

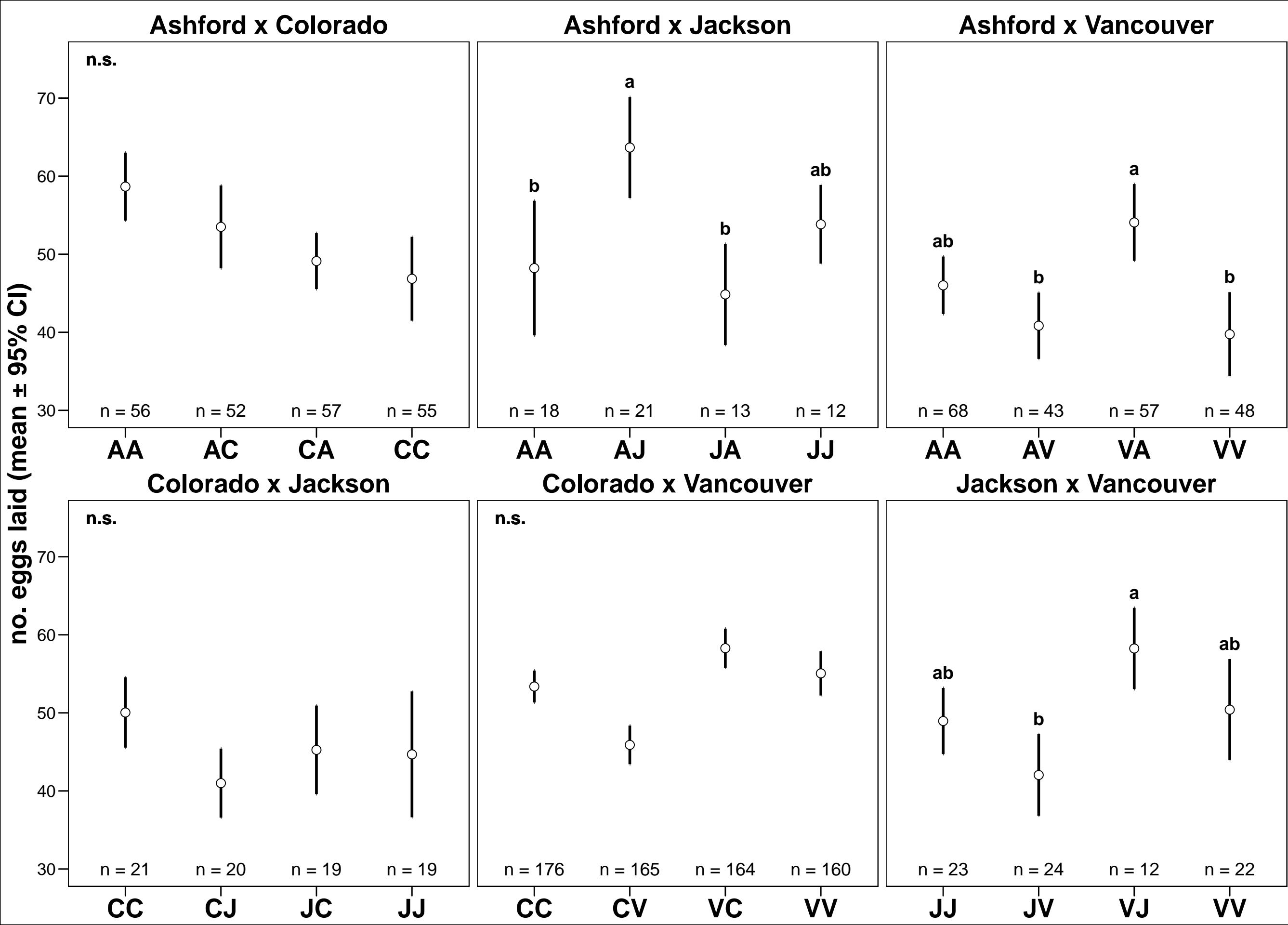

Supplement: Supplementary file 2 [file ECE3-8-9062-s002.pdf]

Ashford x Jackson

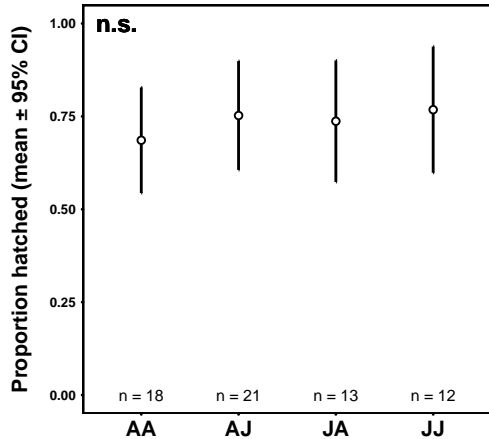

Ashford x Vancouver

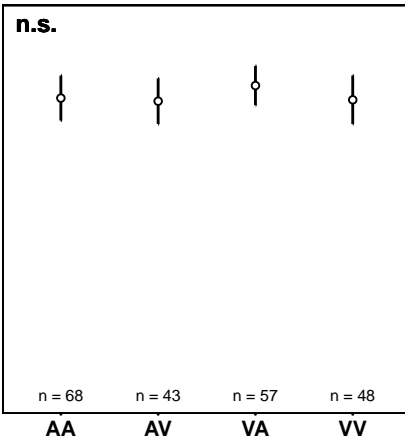

Jackson x Vancouver

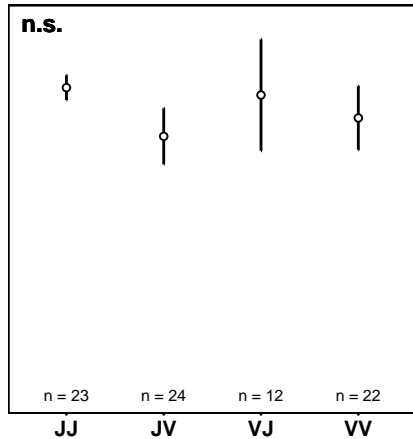

Supplement: Supplementary file 4 [file ECE3-8-9062-s004.pdf]

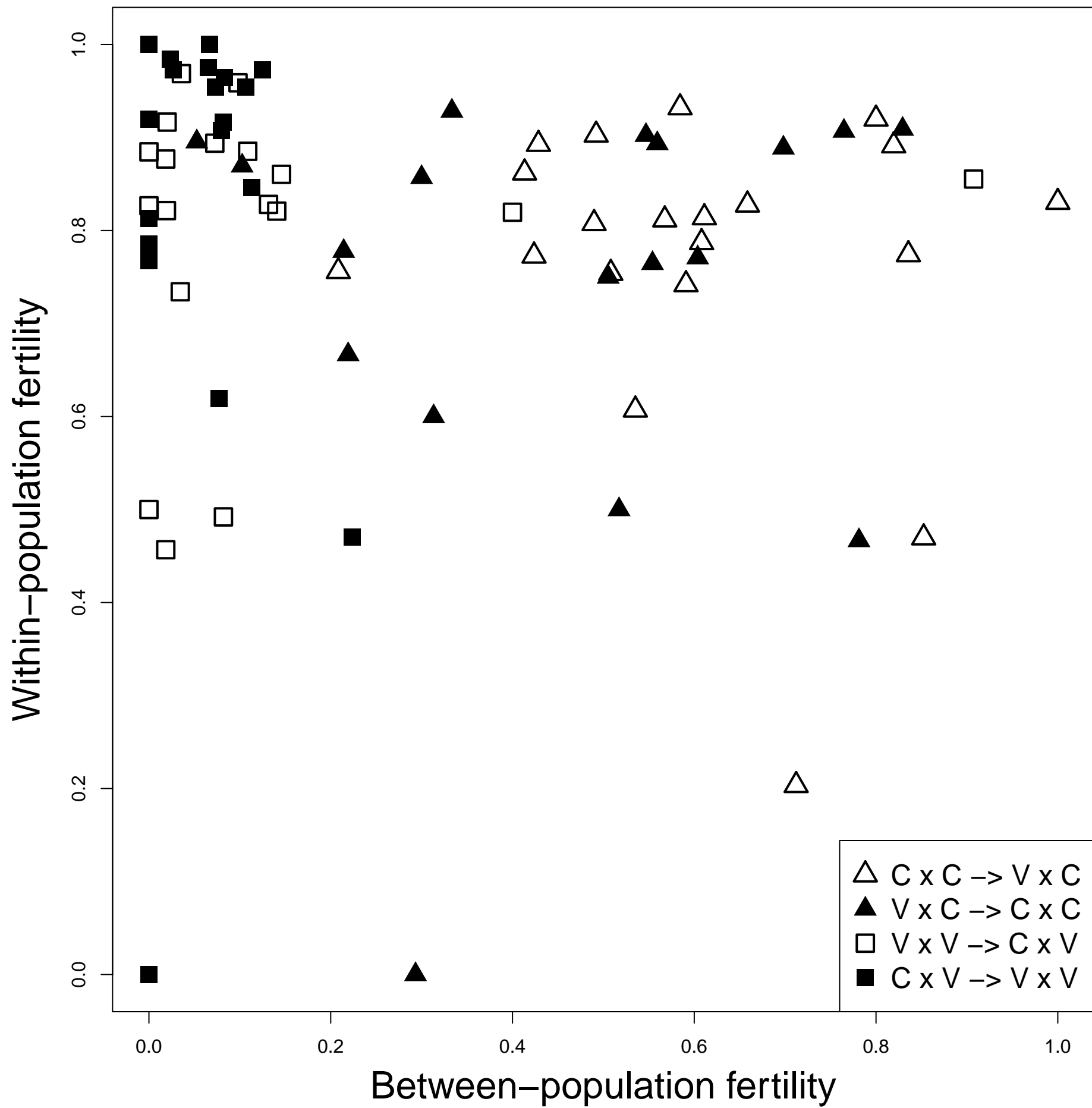

Supplement: Supplementary file 5 [file ECE3-8-9062-s005.pdf]
