# Supplementary material for: Persistent postmating, prezygotic reproductive isolation between populations
Source: Ecol Evol. 2018 Aug 19;8(17):9062–73. doi: 10.1002/ece3.4441 (PMC6157668; doi:10.1002/ece3.4441)
Supplement: Supplementary file 3 [file ECE3-8-9062-s003.pdf]

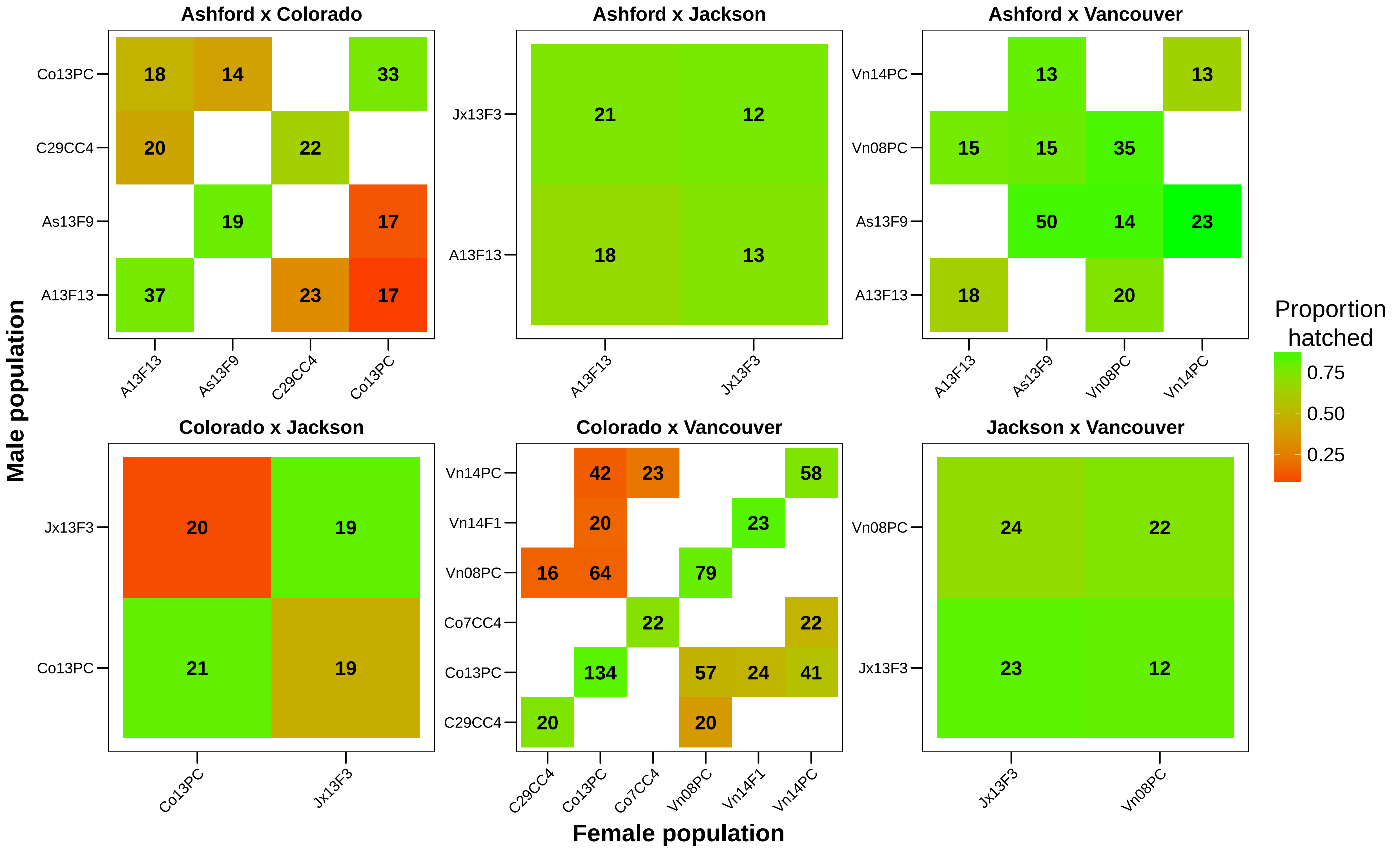
Ashford x Colorado
Co13PC
C29CC4
As13F9
A13F13
18 14 33
20 22
19 17
37 23 17
A13F13
As13F9
C29CC4
Co13PC
Ashford x Jackson
Jx13F3
A13F13
21 12
18 13
A13F13
Jx13F3
Ashford x Vancouver
Vn14PC
Vn08PC
As13F9
A13F13
13 13
15 15 35
50 14 23
18 20
A13F13
As13F9
Vn08PC
Vn14PC
Proportion hatched
0.75
0.50
0.25
Colorado x Jackson
Jx13F3
Co13PC
20 19
21 19
Co13PC
Jx13F3
Colorado x Vancouver
Vn14PC
Vn14F1
Vn08PC
Co7CC4
Co13PC
C29CC4
42 23 58
20 23
16 64 79
22 22
134 57 24 41
20 20
C29CC4
Co13PC
Co7CC4
Vn08PC
Vn14F1
Vn14PC
Jackson x Vancouver
Vn08PC
Jx13F3
24 22
23 12
Jx13F3
Vn08PC
Male population
Female population
