# Supplementary material for: Persistent postmating, prezygotic reproductive isolation between populations
Source: Ecol Evol. 2018 Aug 19;8(17):9062–73. doi: 10.1002/ece3.4441 (PMC6157668; doi:10.1002/ece3.4441)
Supplement: Supplementary file 6 [file ECE3-8-9062-s006.docx]

Table S1. Location and year of collection of *Drosophila montana* strains used in this study.

|  | Location | Coordinates | Altitude (m) | Year | Strain |
| --- | --- | --- | --- | --- | --- |
| Population cages | Crested Butte, Colorado, USA | 38°49’N, 107°04’W | 2868 | 2013 | Co13PC†‡ |
|  | Vancouver, British Columbia, Canada | 48°55’N, 123°48’W | 142 | 2008 | Vn08PC†‡ |
|  |  | 49°11'N, 123°10'W | 4 | 2014 | Vn14PC |
| Iso-female lines | Ashford, Washington, USA | 46°45’N, 121°57’W | 573 | 2013 | As13F9 |
|  |  |  |  | 2013 | A13F13 |
|  | Crested Butte, Colorado, USA | 38°49’N, 107^o^04’W | 2868 | 2009 | Co7CC4 |
|  |  |  |  | 2009 | C29CC4 |
|  | Jackson, Wyoming, USA | 43°26’N, 110°50’W | 1857 | 2013 | Jx13F3‡ |
|  | Vancouver, British Columbia, Canada | 49°15'N, 123°10'W | 4 | 2014 | Vn14F1 |

†Strains used in experiments testing female genotype x male genotype interactions and female and male mating history effects on PMPZ.

‡ Strains used for DAPI staining eggs to confirm prezygotic egg hatch failure.

Table S2. Progeny counts and sex ratio (female:male) in crosses between Co13PC and Vn08PC population cages.

| Cross-type | Total progeny | Total female | Total male | Sex ratio (f:m) |
| --- | --- | --- | --- | --- |
| CC | 815 | 420 | 395 | 1.06 |
| CV | 83 | 32 | 51 | 0.63 |
| VC | 409 | 198 | 211 | 0.94 |
| VV | 582 | 298 | 284 | 1.05 |

Table S3. Post-hoc Tukey’s HSD tests for PMPZ reproductive barriers (fecundity and hatching success) in crosses between Ashford and Colorado.

| ASHFORD X COLORADO | | | | | | | | |
| --- | --- | --- | --- | --- | --- | --- | --- | --- |
|  |  | | Fecundity | |  | |  | |
| Linear Hypotheses: | **Estimate** | | **Std. Error** | | **t value** | | **Pr(>\|t\|)** | |
| AC - AA | -0.041 | | 0.132 | | -0.314 | | 0.989 | |
| CA - AA | -0.071 | | 0.131 | | -0.543 | | 0.948 | |
| CC - AA | -0.238 | | 0.144 | | -1.650 | | 0.350 | |
| CA - AC | -0.030 | | 0.120 | | -0.248 | | 0.995 | |
| CC - AC | -0.197 | | 0.132 | | -1.496 | | 0.439 | |
| CC - CA | -0.167 | | 0.131 | | -1.276 | | 0.577 | |
|  |  | |  | |  | |  | |
| Hatching success | | | | | | | | |
| Linear Hypotheses: | | **Estimate** | | **Std. Error** | | **z value** | | **Pr(>\|z\|)** |
| AC - AA | | -1.993 | | 0.463 | | -4.308 | | **< 0.001** |
| CA - AA | | -3.597 | | 0.462 | | -7.780 | | **< 0.001** |
| CC - AA | | -0.388 | | 0.493 | | -0.788 | | 0.860 |
| CA - AC | | -1.604 | | 0.430 | | -3.728 | | **0.001** |
| CC - AC | | 1.605 | | 0.463 | | 3.467 | | **0.003** |
| CC - CA | | 3.209 | | 0.462 | | 6.950 | | **< 0.001** |

Table S4. Post-hoc Tukey’s HSD tests for PMPZ reproductive barriers (fecundity and hatching success) in crosses between Ashford and Jackson.

| ASHFORD X JACKSON | | | | | | | | |
| --- | --- | --- | --- | --- | --- | --- | --- | --- |
|  |  | | Fecundity | |  | |  | |
| Linear Hypotheses: | **Estimate** | | **Std. Error** | | **t value** | | **Pr(>\|t\|)** | |
| AJ - AA | 0.310 | | 0.106 | | 2.915 | | **0.019** | |
| JA - AA | -0.044 | | 0.122 | | -0.363 | | 0.983 | |
| JJ - AA | 0.159 | | 0.124 | | 1.291 | | 0.567 | |
| JA - AJ | -0.354 | | 0.117 | | -3.022 | | **0.013** | |
| JJ - AJ | -0.150 | | 0.119 | | -1.266 | | 0.583 | |
| JJ - JA | 0.204 | | 0.133 | | 1.532 | | 0.417 | |
|  |  | |  | |  | |  | |
| Hatching success | | | | | | | | |
| Linear Hypotheses: | | **Estimate** | | **Std. Error** | | **z value** | | **Pr(>\|z\|)** |
| AJ - AA | | 0.713 | | 0.726 | | 0.981 | | 0.759 |
| JA - AA | | 0.476 | | 0.817 | | 0.583 | | 0.937 |
| JJ - AA | | 0.625 | | 0.834 | | 0.749 | | 0.876 |
| JA - AJ | | -0.236 | | 0.797 | | -0.296 | | 0.991 |
| JJ - AJ | | -0.088 | | 0.815 | | -0.107 | | 1.000 |
| JJ - JA | | 0.149 | | 0.896 | | 0.166 | | 0.998 |

Table S5. Post-hoc Tukey’s HSD tests for PMPZ reproductive barriers (fecundity and hatching success) in crosses between Ashford and Vancouver.

| ASHFORD X VANCOUVER | | | | | | | | |
| --- | --- | --- | --- | --- | --- | --- | --- | --- |
|  |  | | Fecundity | |  | |  | |
| Linear Hypotheses: | **Estimate** | | **Std. Error** | | **t value** | | **Pr(>\|t\|)** | |
| AV - AA | -0.072 | | 0.084 | | -0.856 | | 0.827 | |
| VA - AA | 0.171 | | 0.077 | | 2.224 | | 0.116 | |
| VV - AA | -0.119 | | 0.082 | | -1.451 | | 0.466 | |
| VA - AV | 0.243 | | 0.087 | | 2.796 | | **0.026** | |
| VV - AV | -0.047 | | 0.091 | | -0.518 | | 0.955 | |
| VV - VA | -0.291 | | 0.085 | | -3.422 | | **0.003** | |
|  |  | |  | |  | |  | |
| Hatching success | | | | | | | | |
| Linear Hypotheses: | | **Estimate** | | **Std. Error** | | **z value** | | **Pr(>\|z\|)** |
| AV - AA | | -0.072 | | 0.084 | | -0.856 | | 0.827 |
| VA - AA | | 0.171 | | 0.077 | | 2.224 | | 0.116 |
| VV - AA | | -0.119 | | 0.082 | | -1.451 | | 0.466 |
| VA - AV | | 0.243 | | 0.087 | | 2.796 | | **0.026** |
| VV - AV | | -0.047 | | 0.091 | | -0.518 | | 0.955 |
| VV - VA | | -0.291 | | 0.085 | | -3.422 | | **0.003** |

Table S6. Post-hoc Tukey’s HSD tests for PMPZ reproductive barriers (fecundity and hatching success) in crosses between Colorado and Jackson.

| COLORADO X JACKSON | | | | | | | | |
| --- | --- | --- | --- | --- | --- | --- | --- | --- |
|  |  | | Fecundity | |  | |  | |
| Linear Hypotheses: | **Estimate** | | **Std. Error** | | **t value** | | **Pr(>\|t\|)** | |
| CJ - CC | -0.204 | | 0.124 | | -1.641 | | 0.355 | |
| JC - CC | -0.112 | | 0.126 | | -0.888 | | 0.811 | |
| JJ - CC | -0.185 | | 0.127 | | -1.463 | | 0.460 | |
| JC - CJ | 0.093 | | 0.128 | | 0.723 | | 0.888 | |
| JJ - CJ | 0.019 | | 0.129 | | 0.147 | | 0.999 | |
| JJ - JC | -0.074 | | 0.130 | | -0.565 | | 0.942 | |
|  |  | |  | |  | |  | |
| Hatching success | | | | | | | | |
| Linear Hypotheses: | | **Estimate** | | **Std. Error** | | **z value** | | **Pr(>\|z\|)** |
| CJ - CC | | -4.156 | | 0.335 | | -12.419 | | **< 0.001** |
| JC - CC | | -1.813 | | 0.316 | | -5.729 | | **< 0.001** |
| JJ - CC | | 0.450 | | 0.332 | | 1.356 | | 0.527 |
| JC - CJ | | 2.344 | | 0.337 | | 6.962 | | **< 0.001** |
| JJ - CJ | | 4.606 | | 0.351 | | 13.119 | | **< 0.001** |
| JJ - JC | | 2.263 | | 0.336 | | 6.744 | | **< 0.001** |

Table S7. Post-hoc Tukey’s HSD tests for PMPZ reproductive barriers (fecundity and hatching success) in crosses between Colorado and Vancouver.

| COLORADO X VANCOUVER | | | | | | | | |
| --- | --- | --- | --- | --- | --- | --- | --- | --- |
|  |  | | Fecundity | |  | |  | |
| Linear Hypotheses: | **Estimate** | | **Std. Error** | | **t value** | | **Pr(>\|t\|)** | |
| CV - CC | -0.137 | | 0.091 | | -1.507 | | 0.432 | |
| VC - CC | 0.109 | | 0.089 | | 1.226 | | 0.609 | |
| VV - CC | 0.032 | | 0.097 | | 0.325 | | 0.988 | |
| VC - CV | 0.247 | | 0.079 | | 3.103 | | **0.010** | |
| VV - CV | 0.169 | | 0.087 | | 1.943 | | 0.209 | |
| VV - VC | -0.078 | | 0.086 | | -0.904 | | 0.802 | |
|  |  | |  | |  | |  | |
| Hatching success | | | | | | | | |
| Linear Hypotheses: | | **Estimate** | | **Std. Error** | | **z value** | | **Pr(>\|z\|)** |
| CV - CC | | -3.655 | | 0.292 | | -12.520 | | **< 0.001** |
| VC - CC | | -1.720 | | 0.285 | | -6.035 | | **< 0.001** |
| VV - CC | | 0.076 | | 0.321 | | 0.237 | | 0.995 |
| VC - CV | | 1.935 | | 0.247 | | 7.827 | | **< 0.001** |
| VV - CV | | 3.731 | | 0.277 | | 13.479 | | **< 0.001** |
| VV - VC | | 1.796 | | 0.275 | | 6.541 | | **< 0.001** |

Table S8. Post-hoc Tukey’s HSD tests for PMPZ reproductive barriers (fecundity and hatching success) in crosses between Jackson and Vancouver.

| JACKSON X VANCOUVER | | | | | | | | |
| --- | --- | --- | --- | --- | --- | --- | --- | --- |
|  |  | | Fecundity | |  | |  | |
| Linear Hypotheses: | **Estimate** | | **Std. Error** | | **t value** | | **Pr(>\|t\|)** | |
| JV - JJ | -0.168 | | 0.093 | | -1.817 | | 0.264 | |
| VJ - JJ | 0.187 | | 0.111 | | 1.690 | | 0.327 | |
| VV - JJ | 0.013 | | 0.094 | | 0.137 | | 0.999 | |
| VJ - JV | 0.355 | | 0.111 | | 3.213 | | **0.007** | |
| VV - JV | 0.181 | | 0.094 | | 1.934 | | 0.212 | |
| VV - VJ | -0.174 | | 0.111 | | -1.562 | | 0.399 | |
|  |  | |  | |  | |  | |
| Hatching success | | | | | | | | |
| Linear Hypotheses: | | **Estimate** | | **Std. Error** | | **z value** | | **Pr(>\|z\|)** |
| JV - JJ | | -0.729 | | 0.296 | | -2.462 | | 0.065 |
| VJ - JJ | | 0.081 | | 0.366 | | 0.220 | | 0.996 |
| VV - JJ | | -0.421 | | 0.302 | | -1.391 | | 0.502 |
| VJ - JV | | 0.810 | | 0.363 | | 2.233 | | 0.113 |
| VV - JV | | 0.309 | | 0.298 | | 1.036 | | 0.726 |
| VV - VJ | | -0.501 | | 0.368 | | -1.363 | | 0.520 |

Table S9. Summary of models testing between-male variance in hatching success after mating between three and five within- or between- population females over consecutive days. Each cross-type was modelled separately as to not artificially inflate the between-group variance. All models included mating day as the only fixed effect. Estimates of random effects variance excluding male identity random effect or observation level random effect (OLRE) in each cross-type are shown and model Akaike information criterion corrected for small sample size (AICc). Cross-types are abbreviated with the female population given first. C, Colorado; V, Vancouver.

| Cross-type | Random effects | AICc | Variance | |
| --- | --- | --- | --- | --- |
|  |  |  | Male | OLRE† |
| CC | Male + OLRE | 320.159 | 0.000 | 0.647 |
|  | Male | 384.475 | 0.056 | - |
|  | OLRE | 317.427 | - | 0.647 |
| CV | Male + OLRE | 251.387 | 0.000 | 0.807 |
|  | Male | 303.678 | 0.154 | - |
|  | OLRE | 248.655 | - | 0.807 |
| VC | Male + OLRE | 234.192 | 0.000 | 0.564 |
|  | Male | 316.843 | 0.179 | - |
|  | OLRE | 230.592 | - | 0.564 |
| VV | Male + OLRE | 343.720 | 0.000 | 1.078 |
|  | Male | 635.476 | 0.186 | - |
|  | OLRE | 340.948 | - | 1.078 |

†Higher OLRE values indicate more overdispersion in the dataset (Harrison, 2015).

Figure S1. Mean number of eggs laid in each specific strain x strain cross-type. Brighter green colours represent higher fecundity, redder colours represent lower fecundity. Numbers in each square show total number of successfully mating pairs in each cross-type over all experimental blocks. See Table S1 for full description of strain abbreviations.

Figure S2. Number of eggs laid (mean ± 95% CI) in each cross between populations. Within each panel different letters above points indicate significant differences from posthoc Tukey’s HSD; letters are recycled in each panel. n.s., non-significant. N = number of mating pairs over all experimental blocks.

Figure S3. Mean proportion of eggs hatched in each specific strain x strain cross-type. Brighter green colours represent higher hatching success; more red colours represent lower hatching success. Numbers in each square show total number of successfully mating pairs in each cross-type over all experimental blocks. See Table S1 for full description of strain abbreviations.

Figure S4. Proportion of eggs hatching (mean ± 95% CI) in crosses not showing PMPZ isolation. n.s., non-significant. N = number of mating pairs over all experimental blocks.

Figure S5. Correlation in fertility (proportion of eggs hatching) between the first and second mating for males mated to both a within- and between-population female. Triangles, Colorado males; Squares, Vancouver males. Open points, first mating = within-population female; filled points, first mating = between-population female.
